# Signaling Downstream of Focal Adhesions Regulates Stiffness-Dependent Differences in the TGF-*β*1-Mediated Myofibroblast Differentiation of Corneal Keratocytes

**DOI:** 10.3389/fcell.2022.886759

**Published:** 2022-05-25

**Authors:** Daniel P. Maruri, Krithika S. Iyer, David W. Schmidtke, W. Matthew Petroll, Victor D. Varner

**Affiliations:** ^1^ Department of Bioengineering, University of Texas at Dallas, Richardson, TX, United States; ^2^ Department of Surgery, UT Southwestern Medical Center, Dallas, TX, United States; ^3^ Department of Ophthalmology, UT Southwestern Medical Center, Dallas, TX, United States

**Keywords:** TGF-*β*1, extracellular matrix, traction force microscopy, FAK, mechanobiology

## Abstract

Following injury and refractive surgery, corneal wound healing can initiate a protracted fibrotic response that interferes with ocular function. This fibrosis is related, in part, to the myofibroblast differentiation of corneal keratocytes in response to transforming growth factor beta 1 (TGF-*β*1). Previous studies have shown that changes in the mechanical properties of the extracellular matrix (ECM) can regulate this process, but the mechanotransductive pathways that govern stiffness-dependent changes in keratocyte differentiation remain unclear. Here, we used a polyacrylamide (PA) gel system to investigate how mechanosensing *via* focal adhesions (FAs) regulates the stiffness-dependent myofibroblast differentiation of primary corneal keratocytes treated with TGF-*β*1. Soft (1 kPa) and stiff (10 kPa) PA substrata were fabricated on glass coverslips, plated with corneal keratocytes, and cultured in defined serum free media with or without exogenous TGF-*β*1. In some experiments, an inhibitor of focal adhesion kinase (FAK) activation was also added to the media. Cells were fixed and stained for F-actin, as well as markers for myofibroblast differentiation (*α*-SMA), actomyosin contractility phosphorylated myosin light chain (pMLC), focal adhesions (vinculin), or Smad activity (pSmad3). We also used traction force microscopy (TFM) to quantify cellular traction stresses. Treatment with TGF-*β*1 elicited stiffness-dependent differences in the number, size, and subcellular distribution of FAs, but not in the nuclear localization of pSmad3. On stiff substrata, cells exhibited large FAs distributed throughout the entire cell body, while on soft gels, the FAs were smaller, fewer in number, and localized primarily to the distal tips of thin cellular extensions. Larger and increased numbers of FAs correlated with elevated traction stresses, increased levels of *α*-SMA immunofluorescence, and more prominent and broadly distributed pMLC staining. Inhibition of FAK disrupted stiffness-dependent differences in keratocyte contractility, FA patterning, and myofibroblast differentiation in the presence of TGF-*β*1. Taken together, these data suggest that signaling downstream of FAs has important implications for the stiffness-dependent myofibroblast differentiation of corneal keratocytes.

## Introduction

The cornea is the transparent tissue at the front of the eye that helps bend light toward the retina. It is composed of three cellular layers—an epithelium, stroma, and endothelium—but the stromal compartment accounts for the bulk of the corneal thickness and contains a highly organized extracellular matrix (ECM) consisting of lamellae of aligned collagen fibrils (Hassell and Birk, 2010; Jester et al., 2010). This ECM endows the tissue, in part, with its transparent optical properties and, in the healthy cornea, is maintained by a population of cells called corneal keratocytes (Nishida et al., 1988; Fini, 1999). Following injury or refractive surgery, however, these cells become activated into a repair phenotype and differentiate into either fibroblasts or myofibroblasts (Jester et al., 1999). The signaling pathways downstream of transforming growth factor-beta 1 (TGF-*β*1) are key to this process (Jester et al., 1996; Jester and Ho-Chang, 2003; Torricelli et al., 2013), and, in some cases, can lead to a protracted myofibroblast response that causes corneal hazing and impaired ocular function (Boote et al., 2012). Corneal wound healing is also associated with changes in tissue stiffness (Raghunathan et al., 2017), and recent work has shown that the mechanical properties of the ECM can regulate the TGF-*β*1-mediated myofibroblast differentiation of corneal keratocytes (Dreier et al., 2013; Petroll and Miron-Mendoza, 2015; Maruri et al., 2020). But it is still unclear how keratocytes sense changes in ECM stiffness and how mechanotransductive signaling mediates stiffness-dependent changes in myofibroblast differentiation.

Corneal keratocytes are connected to the extracellular environment *via* integrin-containing focal adhesions (FAs), which mechanically couple the cytoskeleton to the ECM (Petit and Thiery, 2000). These dynamic, macromolecular assemblies bind to different ECM components and contain specific integrin subunits, as well as a variety of other adapter proteins and signaling molecules (Zaidel-Bar et al., 2007). FAs have also been identified as key mechanosensors, often in a manner that depends on the activity of focal adhesion kinase (FAK) (Schaller and Parsons, 1994; Wang et al., 2001). In several fibroblastic cell types, FAK inhibition disrupts FA assembly and maturation (Katoh, 2017) and inhibits myofibroblast differentiation (Thannickal et al., 2003). Previous work using cultured embryonic fibroblasts, moreover, has shown that changes in FA size can regulate stiffness-dependent changes in myofibroblast differentiation (Goffin et al., 2006). Even so, although cell-ECM interactions are known to influence the behavior of corneal keratocytes in response to a variety of different growth factors, including TGF-*β*1 (Petroll and Miron-Mendoza, 2015; Raghunathan et al., 2017; Maruri et al., 2020), it is unclear if mechanosensing *via* FAs underlies stiffness-dependent differences in the myofibroblast differentiation of corneal keratocytes.

Here, we used a polyacrylamide (PA) gel system to create flexible substrata of varying stiffnesses, which approximate the mechanical properties of either normal (Winkler et al., 2011; Thomasy et al., 2014) or fibrotic (Raghunathan et al., 2017) corneal tissue. These substrata were functionalized with unpolymerized type I collagen, plated with primary normal rabbit keratocytes (NRKs), and used to determine how changes in substratum stiffness affect the size and subcellular distribution of FAs in the presence of TGF-*β*1. These data were combined with quantitative fluorescence microscopy, as well as pharmacological inhibition of FAK, to investigate if FAK activity contributes to stiffness-dependent changes in myofibroblast differentiation.

## Methods

### Fabrication and Functionalization of Polyacrylamide Substrata

Polyacrylamide (PA) gels were fabricated on 30 mm-diameter glass coverslips, as described previously (Maruri et al., 2020). Briefly, a small droplet of unpolymerized PA solution was placed between two surface-treated glass coverslips and allowed to polymerize completely for 30 min under vacuum. The top slide was then removed using fine forceps, and the surface of the PA gel was functionalized with a solution of 50 μg/ml bovine type I collagen (PureCol; Advanced Biomatrix, San Diego, CA) using the heterobifunctional molecule sulfo-SANPAH (Pierce Biotechnology, Rockford, IL). We used a solution of 7.5% (v/v) acrylamide and 3% (v/v) bis-acrylamide to create soft (1 kPa) PA gels, and a solution of 12.5% (v/v) acrylamide and 17.5% (v/v) bis-acrylamide to create stiff (10 kPa) gels (Maruri et al., 2020), which approximate (respectively) the mechanical properties of either normal (Winkler et al., 2011; Thomasy et al., 2014) or fibrotic corneal tissue (Raghunathan et al., 2017).

### Isolating Primary Corneal Keratocytes

Primary corneal keratocytes were harvested from New Zealand white rabbit eyes (Pel-Freez; Rogers, AR), as described previously (Jester et al., 1996; Petroll et al., 2003). Briefly, after removing the corneal epithelium by swabbing with an alcohol pad and scraping the surface of the eye with a sterile surgical blade, we excised corneal buttons using surgical scissors. The endothelium was then removed from each corneal explant using a disposable scalpel. The corneal buttons were incubated overnight at 37°C in digestion media containing 0.5 mg/ml hyaluronidase (Worthington Biochemicals; Lakewood, NJ), 2 mg/ml collagenase (Gibco), and 2% penicillin/streptomycin/amphotericin B (Lonza, Walkersville, MD). Afterward, normal rabbit keratocytes (NRKs) were centrifuge-pelleted, resuspended in medium, plated in T25 culture flasks, and cultured at 37°C in serum-free medium containing DMEM supplemented with 1% RPMI vitamin mix (Sigma-Aldrich; St. Louis, MO), 100 μM nonessential amino acids (Invitrogen; Carlsbad, CA), 100 μg/ml ascorbic acid (Sigma-Aldrich; St. Louis, MO), and 1% penicillin/streptomycin/amphotericin B (Jester and Ho-Chang, 2003; Lakshman and Petroll, 2012). Maintaining the NRKs in defined serum-free conditions preserves their quiescent phenotype until exogeneous growth factors are added to the culture medium (Jester et al., 1996; Jester and Ho-Chang, 2003).

### Cell Culture and Reagents

First-passage NRKs, cultured for 4–5 days in serum-free conditions, were plated at 30,000 cells/ml (6,300 cells/cm^2^) on either functionalized PA substrata or collagen-coated glass coverslips (∼GPa), as described previously (Maruri et al., 2020). To create collagen-coated glass coverslips, untreated circular coverslips (30 mm-diameter) were incubated with a neutralized solution of 50 mg/ml bovine type I collagen at 37°C for 30 min and then rinsed twice with DMEM (Miron-Mendoza et al., 2015; Kivanany et al., 2018). In all experiments, the culture medium was replaced 24 h after plating with either new serum-free medium or with medium supplemented with 5 ng/ml TGF-*β*1 (Sigma-Aldrich; St. Louis, MO). In some experiments, 1 μM focal adhesion kinase (FAK) inhibitor (PF-573228; Sigma-Aldrich; St. Louis, MO) was also added to the culture medium 24 h after plating. This inhibitor interacts with the ATP-binding pocket of FAK and inhibits FAK phosphorylation on Tyr397 (Slack-Davis et al., 2007). A concentration of 1 μM was used, based on previous work showing 80% inhibition of FAK activation (Slack-Davis et al., 2007), as well as preliminary dose-response experiments confirming keratocyte viability. The NRKs were then incubated for 5 days at 37°C in a cell-culture incubator, with an additional media swap after 48 h of culture.

### Immunofluorescence Imaging

After 5 days of culture in growth-factor-containing media, the NRKs were fixed in a solution of 3% paraformaldehyde, washed 3 times with 1 × phosphate-buffered saline (PBS), and permeabilized in 0.3% Triton X-100 in PBS for 20 min. Samples were then blocked with either 2% bovine serum albumin (BSA) fraction V (Equitech-Bio; Kerrville, TX) or 10% goat serum (Gibco) in PBS for 2 h at room temperature. Afterward, the cells were washed twice with PBS and incubated with primary antibody for 2 h at 37°C (or overnight on a shaker at 4°C). The following primary antibodies were used: anti-p-SMAD3 (1D9; 1:100 dilution) (Santa Cruz Biotechnology; Dallas, TX), anti-*α*-smooth muscle actin (1:600 dilution) (Sigma-Aldrich; St. Louis, MO), anti-phospho-myosin light chain (pMLC) 2 (Ser19; 1:200 dilution) (Cell Signaling; Danvers, MA), and anti-vinculin (1:600) (MilliporeSigma; Burlington, MA). After incubation with primary antibodies, samples were washed three times in PBS and incubated in Alexa-Fluor-conjugated secondary antibody (1:200 dilution) (Invitrogen, Carlsbad, CA) and/or Alexa Fluor 594 phalloidin (1:200 dilution) (Invitrogen, Carlsbad, CA). Afterward, the cells were washed three more times, and then incubated with 4′-6-diamidino2-phenylindole (DAPI; 1:1000 dilution) (Sigma-Aldrich; St. Louis, MO) at room temperature for 20 min.

Confocal images of fixed samples were captured using a Zeiss LSM 800, controlled by Zen 2.3 (blue edition) software, and either a 20×, NA 0.8, Plan-Apochromat objective (Zeiss) or a 40 ×, NA 1.3, Oil DIC Plan-Apochromat objective (Zeiss). Morphometric analysis of cell geometry was performed in ImageJ, as described previously (Maruri et al., 2020). Quantification of focal adhesion (FA) size was also conducted in ImageJ using thresholded images of vinculin immunofluorescence.

### Traction Force Microscopy

To perform traction force microscopy (TFM) experiments, fluorescent polystyrene microspheres were suspended within the unpolymerized PA solution (at a density of 0.04% solids) and PA substrata were fabricated as described above. The embedded beads were used as fiducial markers to track gel deformations during time-lapse culture and to compute cell-generated traction forces (Dembo and Wang, 1999; Munevar et al., 2001; Maskarinec et al., 2009; Maruri et al., 2020). Briefly, we fabricated customized multi-well plates containing collagen-functionalized PA substrata, which were plated with NRKs and cultured in cell-culture incubator in either serum-free conditions or in medium containing TGF-*β*1. In some experiments, cells were also treated with PF-573228 to inhibit FAK. After 48 h of culture, the plate was transferred to a humidified stage-top incubator, situated atop a Zeiss AxioObserver 7 microscope, which was equipped with a motorized stage and an ApoTome.2 structured illumination module. Time-lapse phase contrast and epifluorescence images were captured at the top surface of the gel every 30 min for an additional 72 h of culture. (The thickness of the optical section was ∼4 μm) At the end of each experiment, cells were lysed using a 5% solution of Triton X-100 in PBS to capture the undeformed configuration of the gel. Cell-generated traction stresses were then computed using the Particle Image Velocimetry (PIV) and Fourier Transform Traction Cytometry (FTTC) plugins in ImageJ (Martiel et al., 2015).

### Statistical Analysis

Data represent mean ± standard deviation from at least 3 independent experimental replicates. Statistical comparisons were made in Prism 9 (GraphPad; San Diego, CA) using a two-way ANOVA followed by a Tukey post-hoc test, with *p*-values as specified in the figure legends. Relative frequency histograms were generated in Matlab and used to create cumulative frequency plots, which were then compared statistically using the Kolmogorov-Smirnov test in Prism 9.

## Results

### TGF-*β*1-Induced Smad Activity is Independent of Substratum Stiffness

**FIGURE 1:**
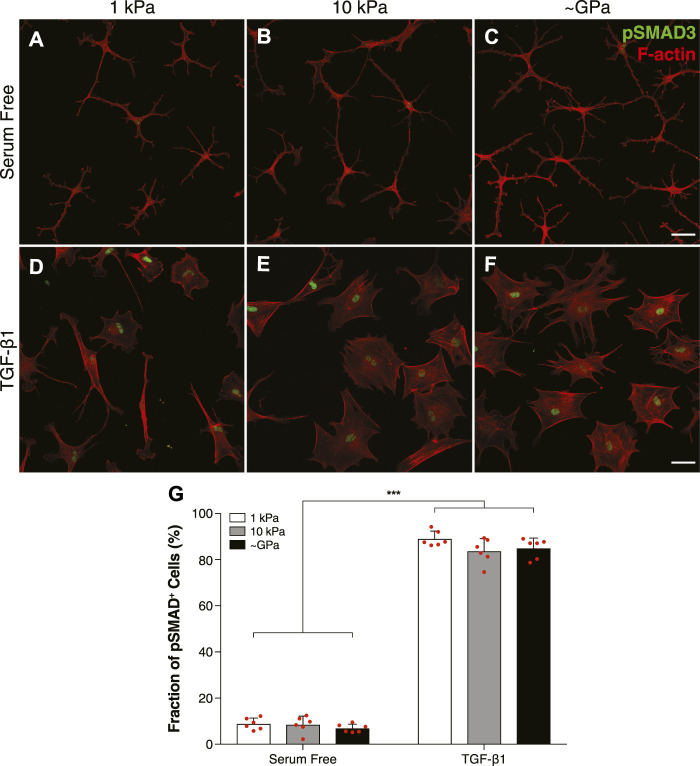
TGF-*β*1-mediated Smad activity is not regulated by substratum stiffness in cultured corneal keratocytes. **(A–F)** Characteristic confocal fluorescence images of cells cultured on either functionalized 1 kPa **(A,D)** or 10 kPa **(B,E)** PA substrata, or collagen-coated glass **(C,F)** coverslips. Prior to fixation, cells were cultured in either serum-free conditions **(A–C)** or medium containing exogenous TGF-*β*1 **(D–F)** for 5 days. Keratocytes were stained for both pSMAD3 immunofluorescence (green) and F-actin (red). **(G)** Bar plots showing the percentage of cells positive for pSMAD3 staining. Error bars represent the mean ± s.d. for *n* = 6 substrates from 3 experimental replicates. A two-way ANOVA with a Tukey post-hoc test was used to evaluate significance among groups. (***, *p* < 0.001). Scale bars, 50 μm.

Previous work in our lab has shown that a soft substratum can decrease levels of myofibroblast differentiation in corneal keratocytes treated with TGF-*β*1 (Maruri et al., 2020). Signaling downstream of TGF-*β*1 activates Smad2/3, which then translocates to the nucleus to promote the expression of genes associated with myofibroblast differentiation (Leask and Abraham, 2004; Hinz, 2007). It is unclear, however, if changes in Smad3 activity underlie the observed stiffness-dependent differences in keratocyte differentiation. To answer this question, we cultured NRKs on substrata of varying stiffnesses and labeled them for pSmad3 immunofluorescence (Figure 1). In serum-free conditions, the NRKs showed negligible levels of pSMAD3 staining on both soft (1 kPa) and stiff (10 kPa) PA substrata, as well as collagen-coated glass coverslips (∼GPa) (Figures 1A–C). In contrast, in the presence of TGF-*β*1, the cells exhibited increased pSMAD3 immunofluorescence, which was localized within cell nuclei (Figures 1D–F). Similar levels of nuclear pSMAD3 staining were observed on substrata of all stiffnesses (Figure 1G), suggesting that changes in ECM stiffness do not modulate Smad activity downstream of TGF-*β*1.

### Substratum Stiffness Modulates the Size and Subcellular Patterning of Focal Adhesions in the Presence of TGF-*β*1

**FIGURE 2:**
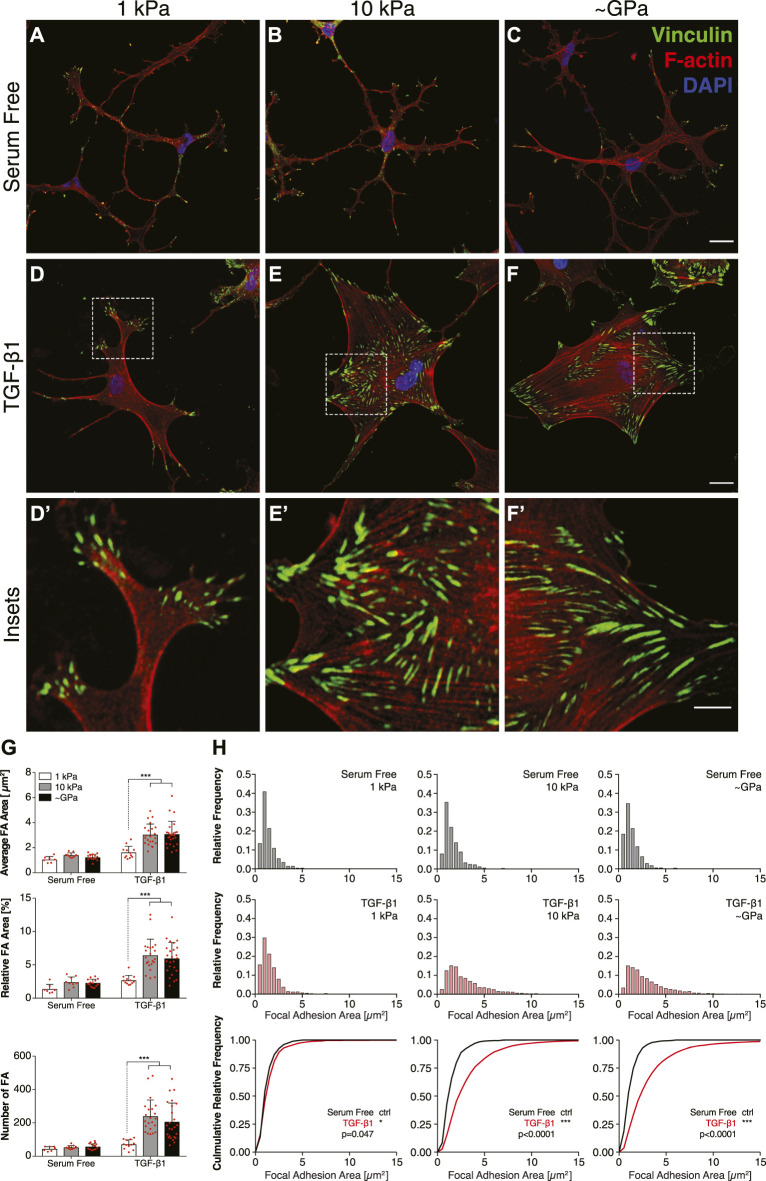
Substratum stiffness modulates the size and subcellular localization of focal adhesions in TGF-*β*1-treated keratocytes. **(A–F)** Characteristic confocal fluorescence images of cells cultured on either functionalized 1 kPa **(A,D)** or 10 kPa **(B,E)** PA substrata, or collagen-coated glass **(C,F)** coverslips. Prior to fixation, cells were cultured in either serum-free conditions **(A–C)** or in medium containing exogenous TGF-*β*1 **(D–F)** for 5 days. Keratocytes were stained for vinculin immunofluorescence (green), as well as F-actin (red) and DAPI (blue). Dashed white boxes indicate insets **(D′–F′)**. Scale bars, 25 μm. (Inset scale bars, 10 μm). **(G)** Quantification of the average area, relative area, and number of focal adhesions (FA) per cell. Error bars represent mean ± s.d. for *n* = 10 substrates from 5 experimental replicates. Statistical comparisons were made using a two-way ANOVA followed by a Tukey post-hoc test. (***, *p* < 0.001) **(H)** Relative frequency histograms of FA area in either serum-free conditions (top row; grey bars) or medium containing TGF-*β*1 (middle row; red bars). These plots were used to create cumulative frequency plots (bottom row), which were compared statistically using a Kolmogorov-Smirnov test.

Cells are connected mechanically to the ECM *via* integrin-containing focal adhesions (FAs), and previous work using different fibroblastic cell types has shown that mechanosensing *via* FAs can regulate stiffness-dependent cell behaviors (Petroll et al., 2003; Goffin et al., 2006). To test this possibility in corneal keratocytes, we stained NRKs cultured on substrata of different stiffnesses for vinculin immunofluorescence and quantified the size and subcellular patterning of FAs (Figure 2). In serum-free conditions, on substrata of all stiffnesses, the cells exhibited a highly branched morphology with cortically localized F-actin and numerous dendritic processes extending outward from the cell body, consistent previous observations (Lakshman et al., 2010) (Figures 2A–C). In each of these cells, the FAs were relatively small and localized primarily at the tips of thin cellular projections (Figures 2A–C). In the presence of TGF-*β*1, however, on both stiff PA substrata and collagen-coated glass coverslips, the cells exhibited a more spread morphology, formed abundant actin stress fibers, and had large focal adhesions, which colocalized with the ends of stress fibers (Figures 2E,F). But on soft PA gels, the TGF-*β*1-treated keratocytes retained a more dendritic morphology, formed fewer stress fibers, and, similar to cells in serum-free conditions, had smaller FAs located within the tips of thin cellular processes (Figure 2D). Quantification of these images revealed significant stiffness-dependent differences in the size and number of FAs in presence of TGF-*β*1, differences that were not present among cells maintained in serum-free conditions (Figures 2G,H), suggesting that changes in substratum stiffness can modulate subcellular patterns of FAs in keratocytes treated with TGF-*β*1.

### Inhibition of Focal Adhesion Kinase (FAK) Disrupts Stiffness-Dependent Differences in Myofibroblast Differentiation

**FIGURE 3:**
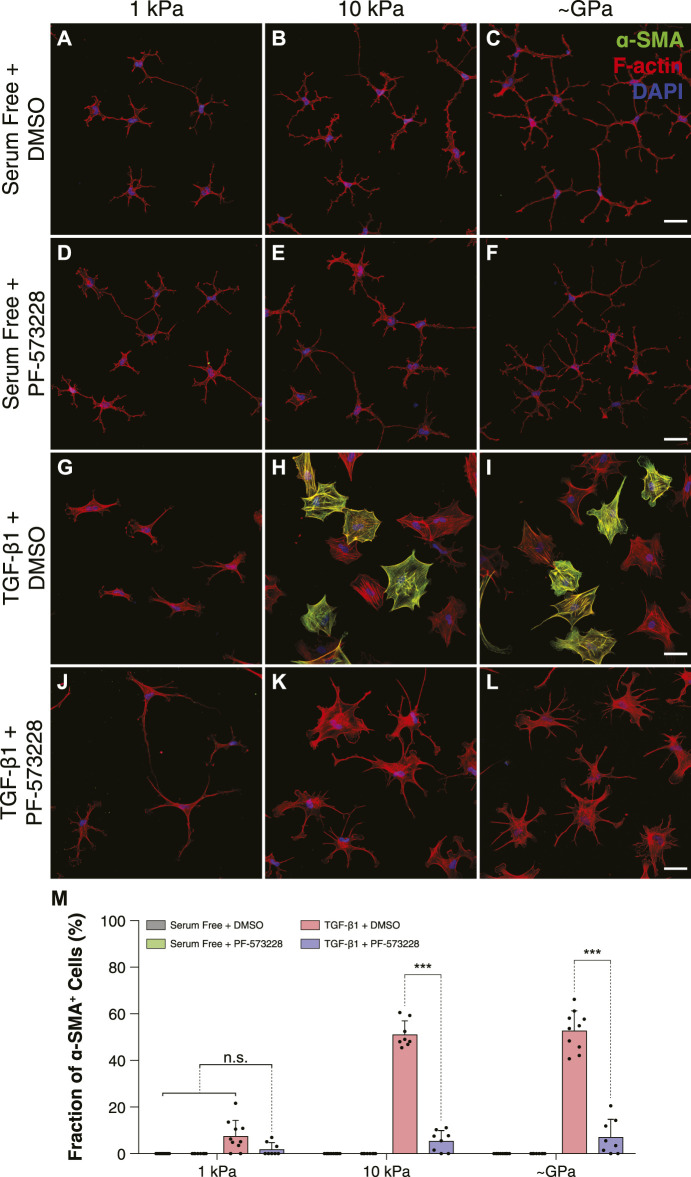
FAK inhibition disrupts stiffness-dependent differences in myofibroblast differentiation in response to TGF-*β*1. **(A–L)** Characteristic confocal fluorescence images of cells cultured on either functionalized 1 kPa **(A,D,G,J)** or 10 kPa **(B,E,H,K)** PA substrata, or collagen-coated glass **(C,F,I,L)** coverslips. Cells were cultured for 5 days in either serum-free conditions **(A–F)** or in medium containing exogenous TGF-*β*1 **(G–L)**, and in either the presence **(D–F,J–L)** or absence **(A–C,G–I)** of the FAK inhibitor, PF-573228. Cells were stained for *α*-SMA immunofluorescence (green), as well as F-actin (red) and DAPI (blue). Scale bars, 50 μm. **(M)** Quantification of the fraction of *α*-SMA-positive cells. Error bars represent mean ± s.d. for *n* = 8 substrates from 4 experimental replicates. A two-way ANOVA with a Tukey post-hoc test was used to evaluate significance among groups. (***, *p* < 0.001).

Previous work has suggested that FA size can influence myofibroblast differentiation in a manner that depends on the activity of focal adhesion kinase (FAK) (Goffin et al., 2006). To determine if FAK activity is involved in the stiffness-dependent differentiation of corneal keratocytes, we cultured TGF-*β*1-treated cells in the presence or absence of the FAK inhibitor PF-573228 and stained them for *α*-SMA immunofluorescence (Figure 3). In serum-free conditions, we observed very few *α*-SMA-positive cells on all substrata (Figures 3A–F). In the presence of TGF-*β*1, however, a substantial number of cells exhibited *α*-SMA staining on either stiff PA substrata or collagen-coated glass coverslips (Figures 3H,I); on soft PA gels, there were significantly fewer *α*-SMA-positive cells (Figure 3G), consistent with previous studies (Dreier et al., 2013; Maruri et al., 2020). When NRKs were treated with both TGF-*β*1 and PF-573228, we no longer observed any stiffness-dependent differences in *α*-SMA staining (Figures 3J–L). Instead on substrata of all stiffness, we observed very few *α*-SMA-positive cells, similar to what was observed in serum-free conditions (Figure 3M).

**FIGURE 4:**
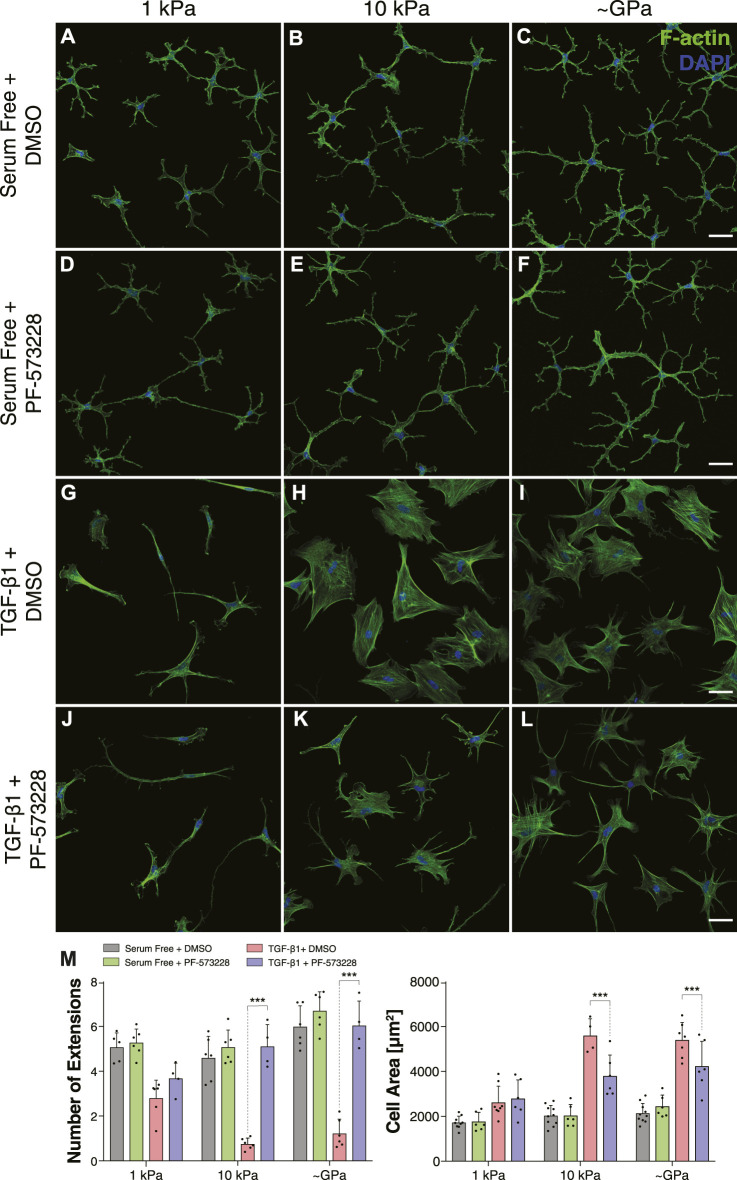
Inhibition of FAK promotes dendritic cell morphologies on substrata of all stiffnesses in the presence of TGF-*β*1. **(A–L)** Characteristic confocal fluorescence images of cells cultured on either functionalized 1 kPa **(A,D,G,J)** or 10 kPa **(B,E,H,K)** PA substrata, or collagen-coated glass **(C,F,I,L)** coverslips. Cells were cultured for 5 days in either serum-free conditions **(A–F)** or in medium containing exogenous TGF-*β*1 **(G–L)**, and in either the presence **(D–F,J–L)** or absence **(A–C,G–I)** of the FAK inhibitor, PF-573228. Cells were stained for both F-actin (green) and DAPI (blue). Scale bars, 50 μm. **(M)** Quantification of cell morphologies. Error bars represent mean ± s.d. for *n* = 6 substrates from 3 experimental replicates. A two-way ANOVA followed by a Tukey post-hoc test was used to evaluate significance among groups. (***, *p* < 0.001).

Inhibition of FAK also disrupted stiffness-dependent differences in keratocyte morphology. In serum-free conditions, NRKs cultured in the presence of PF-573228 were indistinguishable from controls and exhibited the multiple dendritic processes associated with mechanically quiescent keratocytes (Figures 4A–F) (Jester et al., 1994). When treated with TGF-*β*1, as described above, the cultured NRKs had a spread morphology with abundant stress fibers on either stiff PA substrata or collagen-coated glass coverslips but exhibited a more dendritic geometry on soft PA gels (Figures 4G–I). In the presence of PF-573228, however, the TGF-*β*1-treated cells no longer showed stiffness-dependent differences in morphology (Figures 4J–L). Instead, on substrata of all stiffnesses, the keratocytes formed numerous thin cellular projections and showed no evidence of stress fiber formation, behaviors consistent with a more mechanically quiescent phenotype, even in stiff microenvironments (Figures 4J–L). Indeed, on stiff PA substrata and collagen-coated glass coverslips, treatment with PF-573228 elicited an increase in the number of cell extensions, as well as a decrease in cell area in TGF-*β*1-treated keratocytes (Figure 4M).

### FAK Inhibition Restricts Actomyosin Contractility to the Tips of Thin Cellular Extensions

**FIGURE 5:**
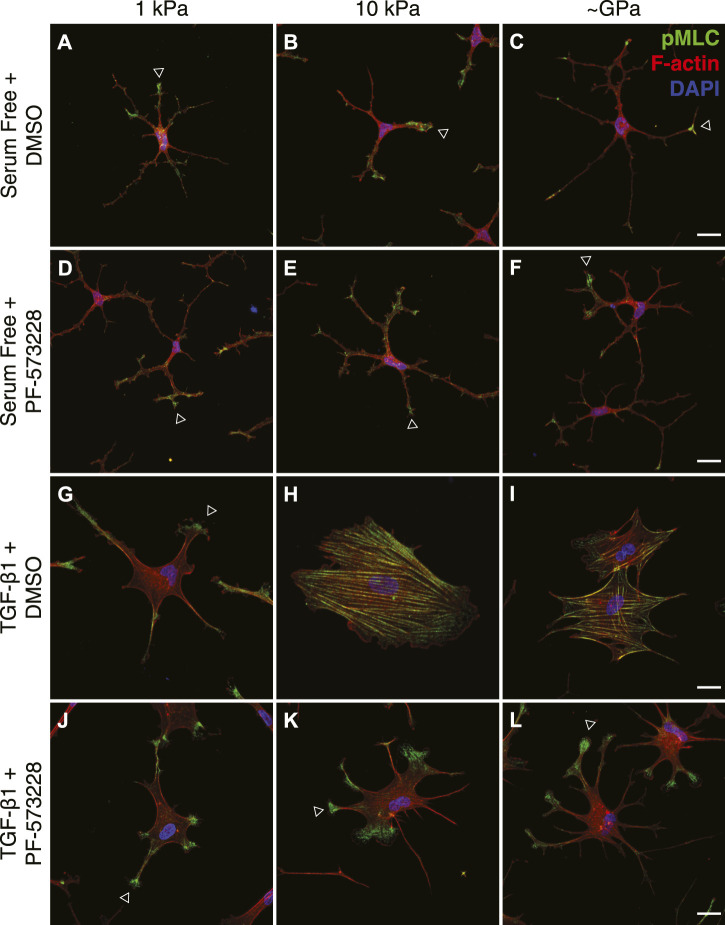
FAK inhibition disrupts stiffness-dependent differences in the subcellular distribution of actomyosin contractility. **(A–L)** Characteristic confocal fluorescence images of cells cultured on either functionalized 1 kPa **(A,D,G,J)** or 10 kPa **(B,E,H,K)** PA substrata, or collagen-coated glass **(C,F,I,L)** coverslips. Cells were cultured for 5 days in either serum-free conditions **(A–F)** or in medium containing exogenous TGF-*β*1 **(G–L)**, and in either the presence **(D–F,J–L)** or absence **(A–C,G–I)** of the FAK inhibitor, PF-573228. Cells were stained for phosphorylated myosin light chain immunofluorescence (pMLC; green), as well as F-actin (red) and DAPI (blue). Arrowheads indicate cellular processes with elevated levels of pMLC staining at the tips. Scale bars, 25 μm.

Myofibroblasts are characterized, in part, by a strongly contractile phenotype (Hinz, 2007; Hinz, 2010), and previous work in our lab has shown that stiffness-dependent differences in the myofibroblast differentiation of corneal keratocytes are associated with distinct subcellular patterns of actomyosin contractility (Maruri et al., 2020). Keratocytes in serum-free conditions exert low contractile forces on substrata of all stiffness, but in the presence of TGF-*β*1, cells cultured on stiff PA substrata exert significantly higher traction stresses than their counterparts on soft PA gels (Maruri et al., 2020). To determine if FAK inhibition alters this stiffness-dependent contractile phenotype, we cultured TGF-*β*1-treated keratocytes on substrata of different stiffnesses in either the presence or absence of PF-573228 and stained them for phosphorylated myosin light chain (pMLC) immunofluorescence (Figure 5). In serum-free conditions, with or without the addition of PF-573228, the observed pMLC staining was weak and localized primarily to the tips of thin cellular extensions, regardless of substratum stiffness (Figures 5A–F). Upon treatment with TGF-*β*1, on stiff PA gels and collagen-coated glass coverslips, pMLC immunofluorescence was present more broadly across the cell body, co-localizing with F-actin-stained stress fibers (Figures 5H,I). On soft PA substrata, however, pMLC staining was similar to that was observed in serum-free conditions, localized primarily within the tips of cellular projections (Figure 5G), consistent with previous observations (Maruri et al., 2020). Treatment with PF-573228 disrupted stiffness-dependent differences in the subcellular localization of pMLC in TGF-*β*1-treated keratocytes (Figures 5J–L). On substrata of all stiffnesses, pMLC was only observed at the tips of cell extensions (Figures 5J–L), similar to NRKs in either serum-free conditions or when cultured on soft PA gels in the presence of TGF-*β*1. This subcellular distribution of pMLC immunofluorescence is associated with a mechanically quiescent keratocyte phenotype (Maruri et al., 2020), suggesting that FAK inhibition disrupts stiffness-dependent differences in NRK contractility.

**FIGURE 6:**
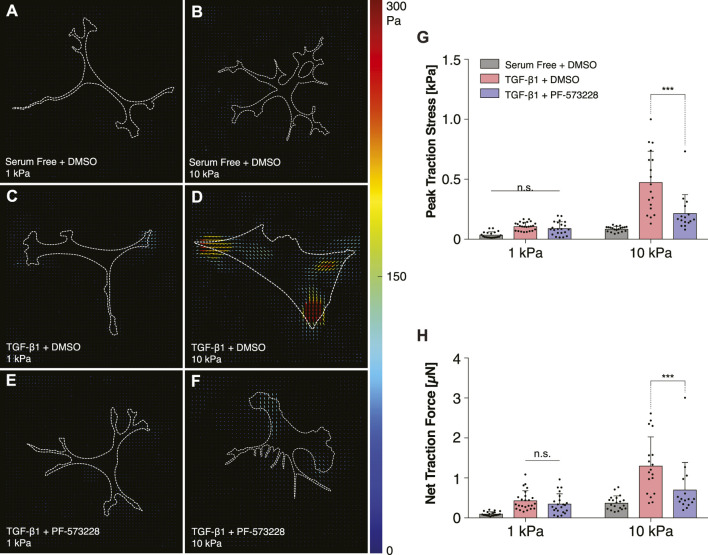
Inhibition of FAK reduces TGF-*β*1-induced traction forces on stiff substrata. **(A–F)** Representative traction stress maps of individual cells cultured on either functionalized 1 kPa **(A,C,E)** or 10 kPa **(B,D,F)** PA substrata. Cells were cultured in either serum-free conditions **(A,B)** or in media containing TGF-*β*1 **(C–F)**, and in either the presence **(E,F)** or absence **(A–D)** of the FAK inhibitor, PF-573228. **(G,H)** Quantification of peak traction stress **(G)** and net traction force **(H)**. Error bars represent mean ± s.d. for at least *n* = 16 cells from 5 experimental replicates. A two-way ANOVA with a Tukey post-hoc test was used to evaluate significance among groups. (***, *p* < 0.001).

Consistently, treatment with PF-573228 also decreased the traction forces generated by TGF-*β*1-treated keratocytes on stiff PA substrata (Figure 6). In serum-free conditions, on both soft and stiff PA substrata, the cultured NRKs exerted very low traction stresses, primarily at the tips of branched cellular projections (Figures 6A,B). In the presence of TGF-*β*1, however, the cells generated greater traction forces on stiff, as opposed to soft, PA substrata (Figures 6C,D,G–H). But in the presence of PF-573228, these stiffness-dependent differences in contractility were significantly reduced (Figures 6E–H). On stiff PA gels, inhibition of FAK produced significant decreases in both peak traction stress and net traction force in cells treated with TGF-*β*1 (Figures 6G,H), a result which further indicates that FAK inhibition can disrupt stiffness-dependent increases in keratocyte contractility.

### FAK Inhibition Blocks Stiffness-Dependent Differences in Focal Adhesion Size and Patterning

**FIGURE 7:**
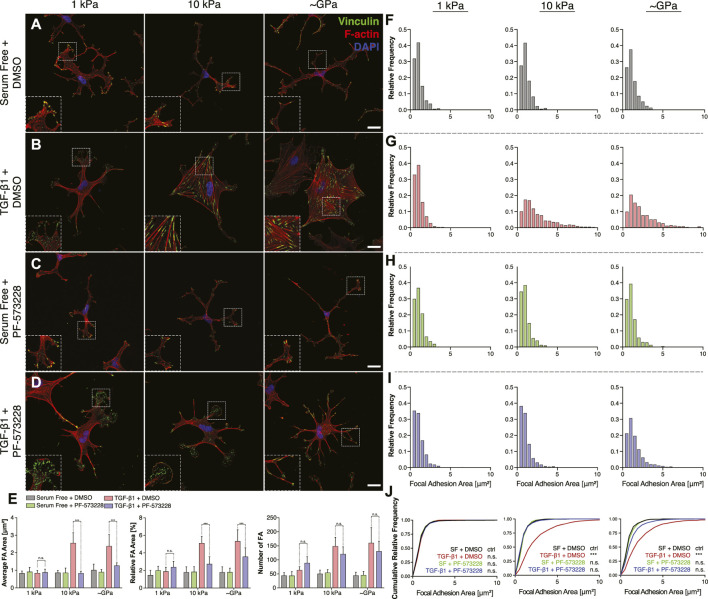
Inhibition of FAK alters subcellular distributions of FAs on stiff substrata in the presence of TGF-*β*1. **(A–D)** Characteristic confocal fluorescence images of primary corneal keratocytes cultured on either soft or stiff PA gels (left or middle columns, respectively), or on collagen coated glass coverslips (right column). Cells were cultured for 5 days in either serum-free conditions **(A,C)** or in medium containing exogenous TGF-*β*1 **(B,D)**, and in either the presence **(C,D)** or absence **(A,B)** of the FAK inhibitor, PF-573228. Cells were stained for vinculin immunofluorescence (green), as well as F-actin (red) and DAPI (blue). Scale bars, 25 μm. Dashed white lines indicate insets. **(E)** Quantification of the average area (left), relative area (middle), and number (right) of focal adhesions (FAs) in individual cells. Error bars represent mean ± s.d. for *n* = 8 substrates from 4 experimental replicates. A two-way ANOVA with a Tukey post-hoc test was used to evaluate significance among groups. (***, *p* < 0.001) **(F–I)** Relative frequency histograms of FA size in cells cultured in either the presence **(H,I)** or absence **(F–G)** of PF-573228. **(J)** Cumulative frequency plots of FA size. A Kolmogorov-Smirnov test was used to evaluate significance between groups.

Treatment with PF-573228 also altered subcellular patterns of FAs within keratocytes cultured in the presence of TGF-*β*1 on stiff substrata. In control experiments, as described above, NRKs formed small FAs on substrata of all stiffnesses when maintained in serum-free conditions (Figure 7A). In the presence of TGF-*β*1, we observed significant increases in FA size and number, with FAs assembling at the termini of F-actin-labeled stress fibers, but only on stiff PA substrata and collagen-coated glass coverslips (Figure 7B). On soft PA gels, the cells exhibited FAs redolent of those observed in serum-free conditions (Figure 7B). When FAK was inhibited using PF-573228, however, we no longer observed stiffness-dependent differences in the size and subcellular patterning of FAs in TGF-*β*1-containing media (Figures 7C,D). Instead, on substrata of all stiffnesses, keratocytes formed small FAs in thin cellular extensions, which were not significantly different from those observed in controls (Figure 7E). Indeed, quantitative analysis of histograms of focal adhesion size revealed that FAK inhibition produced FA distributions that were indistinguishable from controls, even on stiff PA substrata and collagen-coated glass coverslips in TGF-*β*1-containing media (Figures 7F–J). Interestingly, however, the PF-573228-treated keratocytes still formed an increased number of focal adhesions on stiff substrata, even though the FAs were smaller and localized within thin cellular extensions (Figure 7E). Taken together, these data highlight the importance of signaling downstream of FAs during the stiffness-dependent myofibroblast differentiation of corneal keratocytes. Inhibition of FAK disrupts the effects of substratum stiffness, influences the subcellular patterning of FAs, and decreases the contractility and *α*-SMA expression of cultured NRKs.

## Discussion

Cell-ECM interactions have been shown in a variety of cell types to influence myofibroblast differentiation (Hinz et al., 2007; Grinnell and Petroll, 2010; Klingberg et al., 2013; Wells, 2013; Falke et al., 2015; Petroll and Miron-Mendoza, 2015; Zhou et al., 2018). Increases in ECM stiffness promote the expression of *α*-SMA and are thought to underlie a persistent myofibroblast phenotype that can contribute to chronic fibrosis in multiple organs (Marinković et al., 2012; Petroll and Lakshman, 2015; Szeto et al., 2016; Maruri et al., 2020). In many instances, signaling downstream of FAs impacts myofibroblast differentiation (Thannickal et al., 2003), largely *via* changes in the activity of FAK, a molecule that is recruited to FAs in response to mechanical forces (Wang et al., 2001; Martino et al., 2018). FAK activation promotes FA assembly and maturation and initiates downstream mechanotransductive signaling (Ilić et al., 2004; Strohmeyer et al., 2017). In the corneal stroma, the differentiation of corneal keratocytes into myofibroblasts has been shown to depend on ECM stiffness (Dreier et al., 2013; Petroll and Lakshman, 2015; Maruri et al., 2020; Petroll et al., 2020), but it is not known how keratocytes sense changes in the mechanical properties of the ECM and whether signaling downstream of FAs is involved. Inhibition of FAK can disrupt keratocyte differentiation on rigid substrata (e.g., tissue-culture plastic) (Yeung et al., 2021), but it is unclear whether differences in FAK activity are associated with stiffness-dependent differences in keratocyte behavior.

Here, we demonstrated that corneal keratocytes cultured on PA gels of varying stiffness exhibit striking differences in the subcellular localization of FAs when treated with TGF-*β*1. We used soft (1 kPa) gels to mimic the mechanical properties of normal corneal tissue (Winkler et al., 2011; Thomasy et al., 2014), and stiff (10 kPa) gels to approximate the increase in stiffness associated with corneal wound healing (Raghunathan et al., 2017). Consistent with previous studies (Masur et al., 1995; Jester et al., 2003; Petroll et al., 2003), the NRKs cultured on stiff substrata formed large FAs, which were localized to the termini of actin stress fibers that spanned the cell body. On soft PA gels, however, the FAs were smaller, fewer in number, and located primarily within the distal tips of thin cellular extensions. These observations correlated with stiffness-dependent differences in *α*-SMA immunofluorescence, as well as patterns of cellular traction stress (Maruri et al., 2020). Because mechanosensing *via* FAs typically involves the activation of FAK (Chen et al., 2004; Humphrey et al., 2014), we inhibited FAK pharmacologically and disrupted stiffness-dependent differences in myofibroblast differentiation. In the presence of the FAK inhibitor TGF-*β*1-treated keratocytes exhibited extremely low levels of *α*-SMA immunofluorescence and exerted small traction forces on substrata of all stiffnesses. Also, strikingly, changes in substratum stiffness no longer elicited differences in the subcellular pattern of FAs in the presence of TGF-*β*1. Instead, on all substrata FAK inhibition promoted the formation small FAs, localized largely within thin cellular processes—observations consistent a quiescent keratocyte phenotype (Jester et al., 1996; Jester et al., 2003; Maruri et al., 2020). Taken together, these data suggest that FAK activity modulates stiffness-dependent differences in the TGF-*β*1-mediated myofibroblast differentiation of corneal keratocytes.

Previous work using renal fibroblasts has shown that changes in substratum stiffness modulates the phosphorylation and nuclear localization of Smad2/3 downstream of TGF-*β*1, in a manner that involves concomitant changes in Yap activity (Szeto et al., 2016). In cultured corneal keratocytes, however, this appears to not be the case. In the presence of TGF-*β*1, NRKs on all substrata showed nuclear pSmad3 immunofluorescence, even though cells on the softest gels showed very low levels of myofibroblast differentiation. This result suggests that changes in stiffness do not modulate Smad activity downstream of TGF-*β*1, but it remains unclear if cross-talk between Smad- and FAK-dependent pathways also impacts keratocyte behavior. Previous work using TGF-*β*1-treated lung fibroblasts has shown that active Smad signaling occurs in the absence of cell adhesion and FAK phosphorylation (Thannickal et al., 2003). It would be interesting to determine whether a similar mechanism governs the myofibroblast differentiation of corneal keratocytes.

Our data are consistent with previous work that highlights the importance of Rho kinase-dependent contractility during keratocyte differentiation (Chen et al., 2009). FAK activity is intimately tied to cell contraction, since signaling downstream of FAK activates the Rho pathway, which regulates the assembly of actomyosin filaments (Burridge and Chrzanowska-Wodnicka, 1996; Schlaepfer et al., 1999). Inhibition of Rho kinase, moreover, disrupts the contractility and myofibroblast differentiation of corneal keratocytes (Vishwanath et al., 2003; Kim et al., 2006; Chen et al., 2009; Lakshman and Petroll, 2012) and has been shown in other cell types to influence FA assembly (Katoh et al., 2011). In addition, stiffness-dependent changes in FA size, such as those reported here, have been shown to influence a variety of cellular behaviors, such as contractility (Balaban et al., 2001), migration (Kim and Wirtz, 2013), and, in other cell types, myofibroblast differentiation (Goffin et al., 2006; Huang et al., 2012). On polydimethylsiloxane (PDMS) substrata of varying stiffnesses, for instance, cultured rat embryonic fibroblasts exhibit stiffness-dependent differences in the size of FAs and in the recruitment of *α*-SMA to actin stress fibers (Goffin et al., 2006). Interestingly, however, although we reported similar variations in FA size within cultured corneal keratocytes, we also observed a striking stiffness-dependent change in the subcellular distribution of FAs, with cells on softest substrata forming FAs principally within the tips of thin cellular extensions. How these subcellular differences in FA patterning contributes to the mechanotransductive signaling pathways that regulate myofibroblast differentiation remain unclear. Nonetheless, these data highlight the importance of signaling downstream of FAs during the stiffness-dependent differentiation of corneal keratocytes.

## Data Availability

All raw data supporting the conclusions of this article will be made available upon request.
